# A longitudinal study of the subjective birth experience and the relationship to mental health

**DOI:** 10.1186/s12884-025-07348-y

**Published:** 2025-02-27

**Authors:** Sarah Märthesheimer, Carsten Hagenbeck, Martina Helbig, Percy Balan, Tanja Fehm, Nora K. Schaal

**Affiliations:** 1 Department of Experimental Psychology, Heinrich-Heine-University Düsseldorf, Universitätsstraße 1, 40225 Düsseldorf, Germany; 2 Clinic for Gynecology and Obstetrics, University Hospital of Düsseldorf, Moorenstraße 5, 40225 Düsseldorf, Germany

**Keywords:** Childbirth experience, Fear of childbirth, Birth mode, Maternal mental health

## Abstract

**Background:**

A satisfying birth experience has positive effects on the well-being of mother and child. The birth experience depends on subjective expectations and objective birth parameters, and the view of birth can also change over time. However, it is still unclear how birth anxiety and mode of birth affect the different dimensions of the birth experience in the first months after childbirth.

**Methods:**

In this prospective longitudinal study, 307 first-time mothers, planning to give birth vaginally, were assessed for fear of childbirth at approximately 34 weeks of gestation and for obstetric information. Postpartum birth experience and psychological stress was evaluated 2 days, 6 weeks and 6 months postpartum using the validated Childbirth Experience Questionnaire which comprises the four dimensions *emotional experience*, *participation*, *professional support* and *coping possibilitie*s, and a visual analogue scale for a global birth judgement, supplemented by the Edinburgh postpartum depression scale and the Impact of Event Scale.

**Results:**

The individual dimensions of the birth experience changed differently within the first six months. Mixed factorial ANOVAs identified a main effect of fear of childbirth for all four dimensions of the birth experience and the global birth judgment. Mode of birth influenced the dimension *participation* and the global judgement. For *emotional experience*, a complex interplay between fear of birth, birth mode and time was revealed. Correlation analyses showed significant associations between the birth experience and the psychological distress symptoms resulting from childbirth.

**Conclusions:**

Prepartum fear of childbirth affects all dimensions of the subjective birth experience, even after six months. Birth mode, on the other hand, only affects the global birth judgement and participation. The stable correlations between the different dimensions of the birth experience and maternal mental health highlight the importance of the birth experience for clinical practise.

**Trail registration:**

Registered in the German Clinical Trials Register (“DRKS”) (No. DRKS00022177) on 22 June 2020 (https://drks.de/search/en/trial/DRKS00022177).

**Supplementary Information:**

The online version contains supplementary material available at 10.1186/s12884-025-07348-y.

## Introduction

The experience of birth is of great importance for the health of mother and child. Therefore, the WHO recommendation for “intrapartum care for a positive birth experience” emphasises not only a clinical but also a psychologically safe environment [1]. What constitutes a psychologically safe environment for a birth certainly varies from woman to woman [2]. In order to approach this goal, it makes sense to focus on the subjective birth experience of woman giving birth.

Even from a purely physical point of view, the experience of bringing a child into the world is an exceptional experience [3]. The pregnant woman cannot practise or train it. It remains a process with many unknown factors, like the time of the beginning, the exact course and also the outcome. These conditions are also psychologically more or less challenging, at least for some women. This can be seen in the significant proportion of pregnant women who develop a moderate to severe fear of childbirth [4]. Furthermore, some women develop symptoms of post-traumatic stress reactions after a stressful birth, which can lead to a post-traumatic stress disorder [5].

Recent research highlightsfactors which are related to a “successful” birth experience which is satisfying for the mother and, in the best case, for the whole family. From the perspective of the mothers, both external (e.g. birth complications, social support etc.) and internal factors (e.g. psychological states etc.) can play a role. With regard to external conditions, many studies have found links between obstetric complications and maternal satisfaction [6–8]. For example, a recent study showed that a positive birth experience is related to the mode of birth, the duration of birth, the oxytocin augmentation and the use of epidural anaesthesia [9]. A further study showed that women who gave birth vaginally without instrumental support were more satisfied than women who had an operative vaginal birth or a caesarean Sect [6]. The influence of birth pain on the birth experience seems obvious at first, but studies are ambiguous [7, 8, 10–12] and suggest that the influence is rather overestimated [13]. Compared to the influence of the relationship and quality of the accompanying staff and the womans involvement in decisions regarding the birth process, pain and pain reduction were less important [13].

Perceived support during birth plays a crucial role in satisfaction with the birth experience. In order to ensure a positive birth experience, good support during birth seems to be of particular importance [14]. On the other hand, disrespectful behaviour and the lack of social support and/or participation in decision-making is a risk for traumatising birth experiences [7, 12, 15].

Personality traits also affect the birth experience [16]. Anxiety, for example, alters perceived safety, participation and professional support during childbirth. Likewise, women with high levels of neuroticism (personality trait characterized by emotional instability and negative emotions), for example, show less perception of safety during childbirth [17].

A confidence-giving feeling of control can arise when the expectations of birth more or less match the actual experience. If the expectations of labour and birth are fulfilled, there is a higher level of satisfaction with the birth [12]. There seems to be a kind of self-fulfilling prophecy: Ayers and Pickering described in 2005, that women who expected a high level of control in labour, also tended to experience higher control during labour [18]. Another indication of this is the result, that strong prepartum birth anxiety is related to a more negative birth experience reported postpartum [19]. At the same time, expectations are only part of the factors that trigger fear and do not fully describe the fear of childbirth. About 6–15% of women in western countries are affected by severe fear of childbirth (FOC) [4]. FOC during pregnancy is associated with stress, anxiety, depression and a lack of social support and affects first-time and multiparous women [20]. Women with pronounced FOC have a higher risk of obstetric complications, delayed birth and emergency caesarean Sects [21, 22]. These women also more often express the wish for a caesarean section without (further) medical indication [23, 24]. Prepartum FOC is also related to postpartum anxiety levels, and next to other factors, the birth experience and mode of birth in particular play a role to whether high anxiety and fear remain postpartum [22, 25]. There is a kind of vicious circle: women with high FOC tend to have a negative birth experience [26], which in turn leads to renewed stress, strain or anxiety postpartum.

Furthermore, the importance of the subjective birth experience is shown by research highlighting a relationship between the birth experience and the development of a successful mother-child relationship [19], and even the quality of maternal caregiving [27]. There is also evidence from qualitative research that the birth experience is related to infant behaviour. In 18 structured interviews of professional caregivers, Power and colleagues describe that a calmer birth is associated with calmer behaviour of the infant, while physical and emotional stress during birth promoted frequent crying of the baby. According to the authors, the connection exists both directly through the infant’s feeding and indirectly through maternal well-being and the resulting mother-child interaction [28]. Even though this study only very indirectly identifies children’s behaviour through professsional reports, these observations seem to be shared by various caregivers.

Some studies have already shown that the quality of the birth experience is linked to the women’s mental health [29, 30]. The incidence rate of postpartum depression among healthy women is about 17% [31]. Although the correlations are not yet entirely clear, a negative birth experience seems to favour the development of postpartum depression [32]. The subjective birth experience is a significant factor influencing the development of post-traumatic stress symptoms [33, 34]. About 3% of all women even develop a post-traumatic stress disorder as a result of childbirth [35, 36]. The women experience posttraumatic stress symtpoms along with high levels of distress and some of the consequences can be flashbacks, nightmares and anxiety [37]. Posttraumatic stress sysmptoms are associated with poor coping skills and stress, and shows high comorbidity with depression [34, 38]. Here, FOC is also a risk factor. Both, traumatic birth experiences and postpartum depression, influence the development of the mother-child bond [39], highlighting the importance of psychological factors in obstetrics.

Additionally reproductive decisions in the future are influenced by the past birth experience. In a review by Shorey and colleagues in 2018, a positive association between negative birth experience and the decision not to have another child, to postpone a new pregnancy and maternal request for a caesarean section in a subsequent pregnancy is reported [40]. This highlights that a negative birth experience has long-lasting effects on the reproductive behaviour of the women and future obstetric decicions.

The birth experience thus seems to be of great importance for the woman from many perspectives. Very different methods are used to measure *birth experience*, as an overview of available measurement tools from 2017 shows [41]. The comparability of the study results is difficult due to different measurement instruments. Furthermore, the assessment of birth experience (just like memories in general) does not seem to be stable, but changes with the time that has passed since the birth. On the one hand, Stadlmeyer found in a sample that dates back 20 years, that in some parts the birth experience changes within the first two years, while in others it remains stable: women with a low level of perceived intranatal relationship to caregivers and an overall negative birth experience immediately after birth, tended to retain an overall negative experience two years later in all seven (*emotional adaption*,* negative emotional experience*,* physical discomfort*,* fulfilment*,* control*,* anxious and time-going-slowly*) dimensions considered [42]. Waldenström also found particular changes for women with negative birth experiences regarding pain recall [43]. On the other hand, Conde found continuity in the assessment of the birth experience within the first six months postpartum in a rather small sample (*N* = 68) [44].

Due to the complexity of the birth experience, both one-dimensional measures, such as a global assessment, and multidimensional measures should reflect the versatility of the experience. Given the consequences that a negative birth experience can have, it makes sense to examine more closely how these specific aspects of the birth experience develop, whether they remain stable or change over time. Turkmen used the *Childbirth Experience Questionnaire* (CEQ) [45], which revealed four subscales of birth experience, in 2018 for this purpose [46]. Turkmen and colleagues found a reduction in the subscales *professional support* and *participation* within the first three months postpartum, while the general birth satisfaction of the 63 Swedish participating women did not change [45]. For far-reaching conclusions, however, the results must also be viewed critically due to the rather small sample and the occurrence of ceiling effects.

The aim of the present analysis was to systematically examine the different facets of the birth experience in a large sample of women aiming to give birth vaginally and to explore the development of the subjective birth experience over time until 6 months postpartum. Due to the high significance for the birth experience, the influence of FOC was included in the analysis. It is likely that the perceived changes over time in the remembered birth experience are also be influenced by prepartum FOC. In addition, the birth mode was included as a further factor. Because the desire for a sense of control over birth is also significant, the mode of birth may also play a role in the subjective birth experience. In a second step, the connection between the different facets of the birth experience and depressive and traumatic symptoms were examined. Although a link between birth assessment and development of depressive and post-traumatic stress symptoms is suggested by the literature [33], it would be important to understand which aspects of the birth experience are of particular importance in this regard and and how these connections develops over time. The aim is to investigate the change in the birth experience over time and the influence of FOC and birth mode on the birth experience. In addition, the connection between the various birth dimensions and depressive and post-traumatic symptoms will be investigated.

## Methods

### Participants

**Fig. 1:**
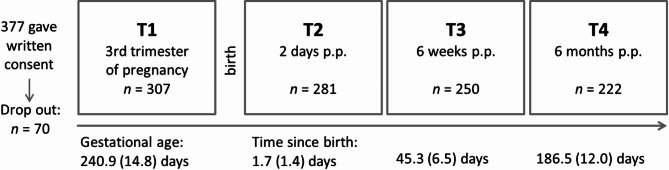
Course of the study. Overview of the one prepartum and three postpartum (p.p.) measurement time points with the respective sample size and actual mean of measurement time (standard deviation in brackets)

All women with a minimum age of 18 years, pregnant for the first time without any severe previous illness and who were planning to give birth vaginally were eligible to participate. Furthermore, speaking a sufficient level of the German language was necessary in order to fill out the questionnaire. Participation in the study was offered when registering for birth at the hospital in the last trimester of pregnancy. 398 women, who met the inclusion criteria, were approached at the Clinic for Gynaecology and Obstetrics at the University hospital in Düsseldorf Germany between July 2020 and November 2021. 21 of 398 did not participate because they did not fulfil the inclusion criteria after all (*n* = 16) or were not interested in participating (*n* = 5)., 377 participants gave informed written consent prior to participation. Seventy women were excluded during the study progress because they did not fill in the first questionnaire (*n* = 13), they gave birth at another hospital (*n* = 35) or they received a planned caesarean section (CS) which was not yet known when women were recruited (*n* = 22). After excluding these cases the final analysis is based on the sample of 307 data sets. As this is a prospective longitudinal study with four measurement time points, sample sizes vary depending on the time of measurement, as can be seen in Fig. 1.

To calculate the necessary sample-size, the program G*Power was used [47]. An a priori power analysis to calculate the required sample size was based on a mixed factorial ANOVA with an estimated effect size of 0.2, an alpha error of 0.05, and a power of at least 0.80, resulting in a required sample size of at least 144 complete participants. The increased final sample size resulted from an originally higher calculation for drop-out due to planned caesarean sections and for drop-out in the later measurement time points. The study complies with the STROBE guidelines.

### Material and measures

Standardised questionnaires and visual analogue scales (VAS) were used for the study. Prepartum, the participants were given one set of questionnaires (T1: 34th week of pregnancy), postpartum they received the same set of questionnaires at three time points (T2: 2 days, T3: 6 weeks and T4: 6 months after birth).

### Antenatal measures (T1)

In order to receive broad and detailed information on birth anxiety, the *Fear of Birth Scale* (FOBS) and the *Wijma Delivery Expectancy Questionnaire* (WDEQ) were used to assess specific pre-birth anxiety. The frequently used WDEQ [48] measures birth expectations with a focus on fears regarding childbirth. It consists of 33 statements about possible sensations or evaluations before and during birth, which can be agreed to on a 6-point-likert scale. The sum score ranges from 0 to 165 points, with higher scores indicating greater fears. The work by Wjima and colleagues suggests three levels: up to 84 points: no significant fear of childbirth, 85 to 99 points: severe childbirth-anxiety, and 100 or more points: phobic childbirth-anxiety [49]. The cut-off score of 85 points is highly used in the literature [4], Chronbach’s alpha is α = 0.91. The FOBS includes two VAS [50] with the question “How do you feel about the approaching birth?” and the anchors “calm – worried” and “no fear – strong fear” respectively. Like all VAS they had a line length of 100 mm on which the participant could tick her degree of agreement between the two anchors which were placed on the right and left. A mean score of more than 60 mm was defined as FOC [51] The internal cosistency is α = 0.91. Demographic data such as maternal age, education level, financial situation and whether the pregnancy resulted after fertility treatment were also collected.

### Postpartal measures (T2, T3, T4)

Birth experience was determined by a one-dimensional global satisfaction using a VAS (VAS overall birth judgement) and by a multidimensional instrument using the Childbirth Experience Questionnaire (CEQ). The German version of the CEQ contains 18 (English original: 22) items with a 4-point-likert-scale (from 1 = totally agree to 4 = totally disagree). The German and the English versions differ in the selection of items used and the subscales determined. For the German version used in this study, subjective birth-experience was measured by calculating the mean scores for the four subscales *emotional experience* (3 items), *coping possibilities* (4 items), *professional support* (8 items) and *participation* (3 items), whereby higher values stand for a stronger confirmation. Internal consistency (calculated for each timepoint) is good and lies between 0.79 ≤ α ≤ 0.85 for *emotional experience*, 0.79 ≤ α ≤ 0.85 for *coping possibilities*, 0.83 ≤ α ≤ 0.91 for *professional support* and 0.64 ≤ α ≤ 0.76 for *participation*. Additionally, a global assessment of birth (with the anchors negative to positive) was recorded by using a VAS (VAS *overall birth judgement*).

The possible consequences of a stressful birth experience were assessed with the German version of the *Edinburgh Postpartum Depression Scale* (EPDS) [52, 53] and the *Impact of Event Scale* (IES) [54, 55]. The frequently used EPDS includes 10 items on a 4-point-likert-cale (0–3) such as low mood, feelings of guilt or overwhelm, or thoughts of self-harming behaviour and records a total score between 0 and 30 points. With 15 items on a 4-point-likert-scale the IES measures whether the birth experience has left post-traumatic stress symptoms, like strong feelings about the memories of the birth, sleep disturbances or avoidance of memory. For both questionnaires, EPDS and IES, higher scores represent more and/or stronger symptoms. The internal consistency (calculated for each timepoint) for the IES is between 0.84 ≤ α ≤ 0.85 and for the EPDS α = 0.83.

### Obstetric measures

Obstetrical information was taken after birth from the medical record. The mode of birth, use of epidural or general anaesthesia, possible induction of labour, amniotomy or micro blood tests sub partu or necessary transfer of the baby to the paediatric clinic were determined.

### Procedure

Women were approached when registering for giving birth at the clinic approximately 6 weeks prior to their expected due date. After the inclusion criteria had been checked and before enrolment, the participants received information about the study and gave their written consent. If the inclusion criteria were not met or at least one exclusion criterion was present, participation was excluded. Afterwards, the women filled out the first questionnaire (T1) in the clinic. About 1–2 days after birth, the participants completed the second questionnaire (T2) on the maternity ward. The maternal and obstetric information was taken from the patient’s electronic file shortly after birth. The first two questionnaires were based on paper-pencil. The following measurements were carried out online, 6 weeks (T3) and 6 months (T4) after birth. For the online-survey the online platform SoSciSurvey [56] was used. Participants were contacted via email at the predetermined time points. The email included a personal ID which served as an entry code to the online questionnaire and which enabled us to merge the data of the four time points. They were asked to complete the questionnaire within one week on their technical devices at home. If the questionnaire remained unanswered, the participants were reminded after one week by email and after another week by a text message on their mobile phone. As a thank you for their participation the women received a baby suit.The evaluation of the data and the writing of the manuscript took place at the neighbouring university. The authors did not have access to information that could identify individual participants after data collection. The study was prospectively approved by the ethics committee of the Medical Department of the Heinrich- Heine-University in Düsseldorf (No. 2020 − 923) on 05.06.2020.

### Statistical analyses

For the statistical analysis the statistical package IBM SPSS Statistics 27 was used. First, the descriptive statistics of the survey as well as the basic medical data were determined. Chi²-tests were calculated to compare the medical outcome between the women with high and low fear of childbirth. The group determination of women with high and low fear of childbirth was calculated based on the mean of FOBS-scales greater or equal 60 mm vs. less 60 mm, as Ternström and colleagues have already done [51]. There was a strong positive correlation between the two FOC measurement tools evaluated at T1; the WDEQ and the FOBS (Pearson correlation: *r* (294) = 0.62, *p* <.001), so that in the further analysis FOC was only based on the FOBS [57]. Additionally, in order to investigate the influence of birth mode on birth experience, the two groups *vaginal birth* (VB: spontaneous parturition and instrumental birth) and *unplanned caesarean section* (CS) were formed.

For the main analysis regarding the birth experience, five mixed-factor 2 × 2 × 3 - ANOVAs were calculated with the between-subject factor FOC (high FOC vs. low FOC), the between-subject factor birth mode (VB: vaginal birth vs. CS: caesarean section) and the within-subject factor time (T2: 2 days vs. T3: 6 weeks vs. T4: 6 months postpartum). The four CEQ subscales *emotional experience*, *participation*, *professional support*, *coping possibilities* and the *VAS scale overall birth judgement* served as dependent variables respectively for the five ANOVAs. Using t-tests (two-tailed) for independent samples, the groups of women with high vs. low FOC and women after vaginal birth vs. caesarean section are tested for mean differences at the different measurement times. If the sphericity assumption was violated, Greenhouse-Geisser corrected values are reported. Bonferroni-corrected post hoc tests are reported.

In the next step, two repeated-measure ANOVAs were calculated to examine the course of depressive symptoms (EPDS) and traumatic symptoms (IES) in order to explore changes over time between T2, T3 and T4. Aditionally, pearson correlations between the subjective birth experience, i.e. the four CEQ scales and the *VAS overall birth judgement* on the one hand and depressive (EPDS) and posttraumatic symptoms following childbirth (IES) on the other hand were calculated.

According to Cohen [57], the limits for the effect size of pearson correlations are 0.10 (small effect), 0.30 (medium effect) and 0.50 (large effect), and the limits for the effect size of ANOVAs were 0.01 (small effect), 0.06 (medium effect) and 0.14 (large effect).

## Results

### Group characteristics & descriptive statistics

The number of participants varied depending on the time of measurement. 72.3% (*N* = 222) of the recruited women completed all time points. T2 questionnaires answered later than 7 days postpartum and T3 and T4 questionnaires answered later than 3 weeks after the first invitation were not included. The absolute number of questionnaires for the four measurement points can be seen in Fig. 1.

The final sample consisted of 307 women expecting their first child with a mean age of 32.9 years (*SD* = 4.4, range: 20 and 49 years). The mean gestational age at T1 was 35 weeks (*M* = 240.9 days, *SD* = 14.8 days; range: 28th to 39th week of pregnancy). At birth, the gestational age of the participants was 40 weeks (*M* = 279.2 days, *SD* = 10.8 days, range 259–295 days).

19.3% (*N* = 58) of the participants showed high FOC with a mean of the FOB scales equal or higher than 60 mm and are assigned to the *high FOC-group* in the following. The remaining 80.7% (*N* = 243) women whose scores were below this threshold were assigned to the group with no or low fear of childbirth and were called the *low FOC-group* in the further course [51]. The descriptive statistics of birth experience (CEQ and *VAS overall birth judgement*) and mental distress (IES and EPDS) were listed in Table 1.

**Table 1:** Descriptives of birth experience and mental distress

	T2 2 days *p*.*p*.		T3 6 weeks *p*.*p*.		T4 6 months *p*.*p*.	
	*N*	*M* (*SD*)	*N*	*M* (*SD*)	*N*	*M* (*SD*)
CEQ *emotional experience*	269	2.49 (0.78)	248	2.59 (0.79)	222	2.66 (0.81)
CEQ *coping possibilities*	269	2.54 (0.70)	248	2.74 (0.68)	222	2.75 (0.71)
CEQ *professional support*	269	3.65 (0.46)	248	3.47 (0.57)	222	3.39 (0.62)
CEQ *participation*	269	3.14 (0.74)	248	3.03 (0.80)	222	3.00 (0.82)
VAS overall birth judgement	278	64.51 (24.84)	250	68.02 (26.02)	222	70.20 (26.10)
IES	274	13.64 (10.97)	249	10.23 (10.11)	219	8.66 (9.35)
EPDS	277	6.22 (5.01)	250	6.40 (4.42)	221	5.55 (4.22)

*Note*: Descriptive statistics of the birth experience(four Childbirth experience questionnairesubscales,range 1–4,and Visual analogue scale *overall birth judgement*, range 0-100, both with higher values for greater satisfaction) and mental distress (Impact of Event scale, range 0–75 and Edingburgh Postpartum Depression Scale, range 0–30, both with higher values for more traumatic or depressive symptoms) for the first 6 months postpartum

The medical parameters arelisted in Table 2. 14.7% (*N* = 45) of the participants became pregnant after fertility treatment. 68.1% (*N* = 209) of the participants gave birth vaginally as aspired, whereas 31.9% (*N* = 98) received a secondary CS. There were few differences in the obstetric parameters between women with low and high FOC: Pearson chi²-tests identified differences in birth mode and the proportion of children transferred to the paediatric clinic after birth. A 2 × 2 - chi²-test showed an association between FOC and birth mode: χ² (1, *N* = 301) = 5.22, *p* =.022: Women in the *high FOC-group* delivered more often by unplanned CS (*n* = 26, 44.8%) than women in the low FOC group (*n* = 71, 29.2%). In addition, proportionally more babies of the *high FOC-group* were transferred to the paediatric clinic after birth (*n* = 6, 10.7%), than babies of the *low FOC-group* (*n* = 9, 3.8%): χ² (1, *N* = 294) = 4.50, *p* =.034. There was a marginal difference between the two groups in the frequency of prematurity (χ² (1, *N* = 298) = 3.45, *p* =.063) and microblood tests during birth (χ² (1, *N* = 295) = 2.87, *p* =.090). With regard to all other listed interventions and complications, there were no more differences between women with high and low FOC (*p* >.178).

**Table 2:** Medical information for the complete sample and for high- vs. low FOC-group

	Full sample	Low FOC-group	High FOC-group	*p*
	*N* (%)	*n* (%)	*n* (%)	
	307 (100%)	243 (80.7%)	58 (19.3%)	
Fertility treatment	45 (14.8%)	35 (14.4%)	9 (15.5%)	0.801
Preterm birth	13 (4.4%)	8 (3.3%)	5 (8.9%)	0.063
Labour induction	93 (30.3%)	71 (29.2%)	21 (36.2%)	0.299
Caesarean section	98 (31.9%)	71 (29.2%)	26 (44.8%)	0.022*
Epidural anaesthesia	197 (65.7%)	153 (64.3%)	42 (72.4%)	0.178
Microblood test	33 (11.0%)	23 (9.7%)	10 (17.2%)	0.090
Transfer to Paediatric clinic	15 (5.0%)	9 (3.8%)	6 (10.7%)	0.034*
Rupture of the membranes	35 (11.7%)	27 (11.3%)	8 (14.3%)	0.541

Note: Absolute numbers and percentages of important (pre-) birth interventions for the whole sample as well as for women with high and low Fear of Childbirth and *p*-values of pearson chi²-test (*p <.05). The microblood test is an intervention during birth: capillary blood was taken vaginally from the foetus’ head for blood gas analysis to assess the baby’s metabolic status

### Birth experience and the influence of time, fear of childbirth and birth mode

**Fig. 2:**
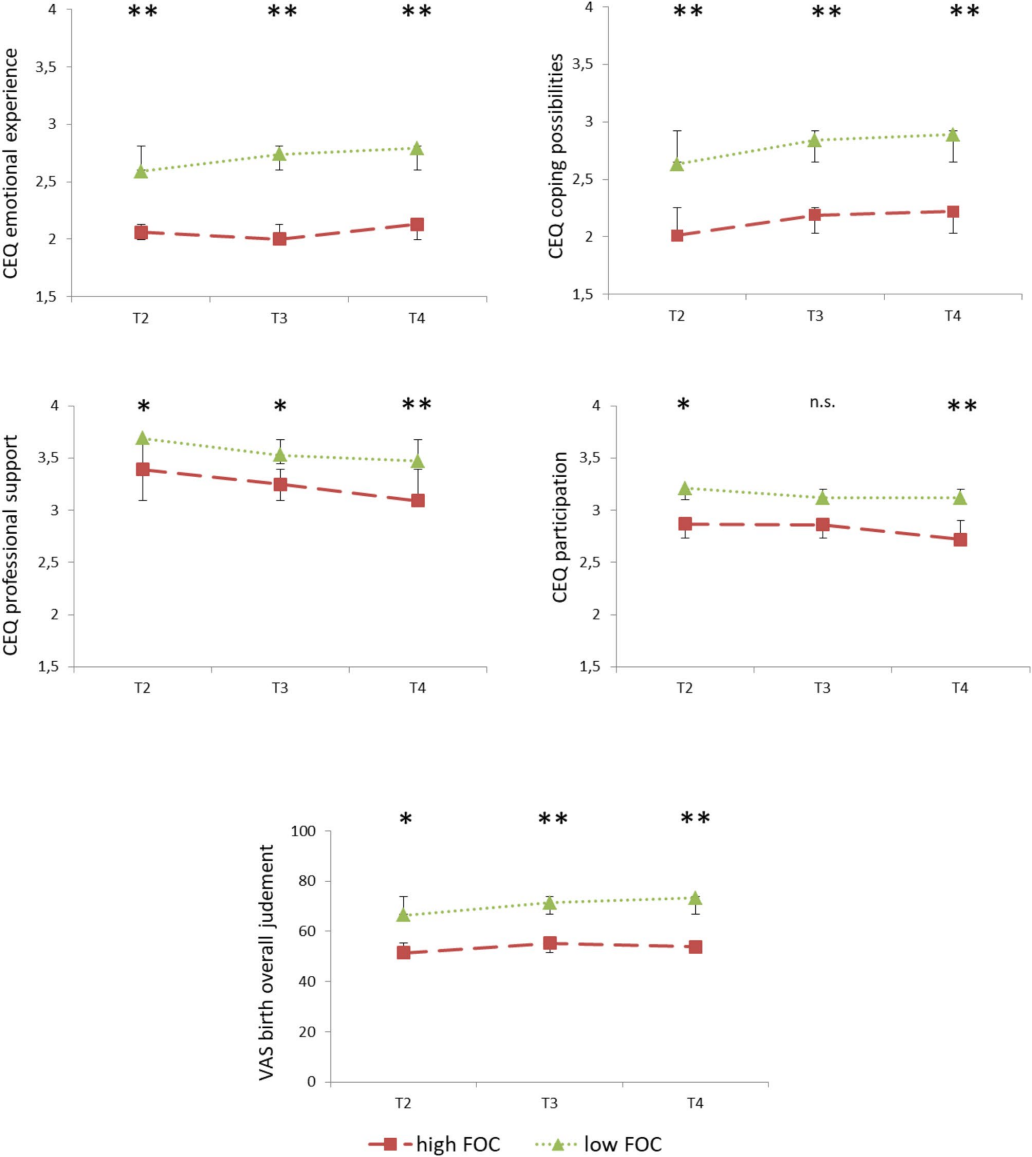
Fear of Childbirth and the course of birth experience. Course of the different birth experiences (Childbirth Experience Questionnaire and Visual Analogue Scale *overall birth judgement*) for high vs. low FOC-goup with standard deviations. The asterix show the results of the t-tests (two-tailed) for independent samples between high and low FOC (*p <.05, ** *p* <.001)

**Fig. 3:**
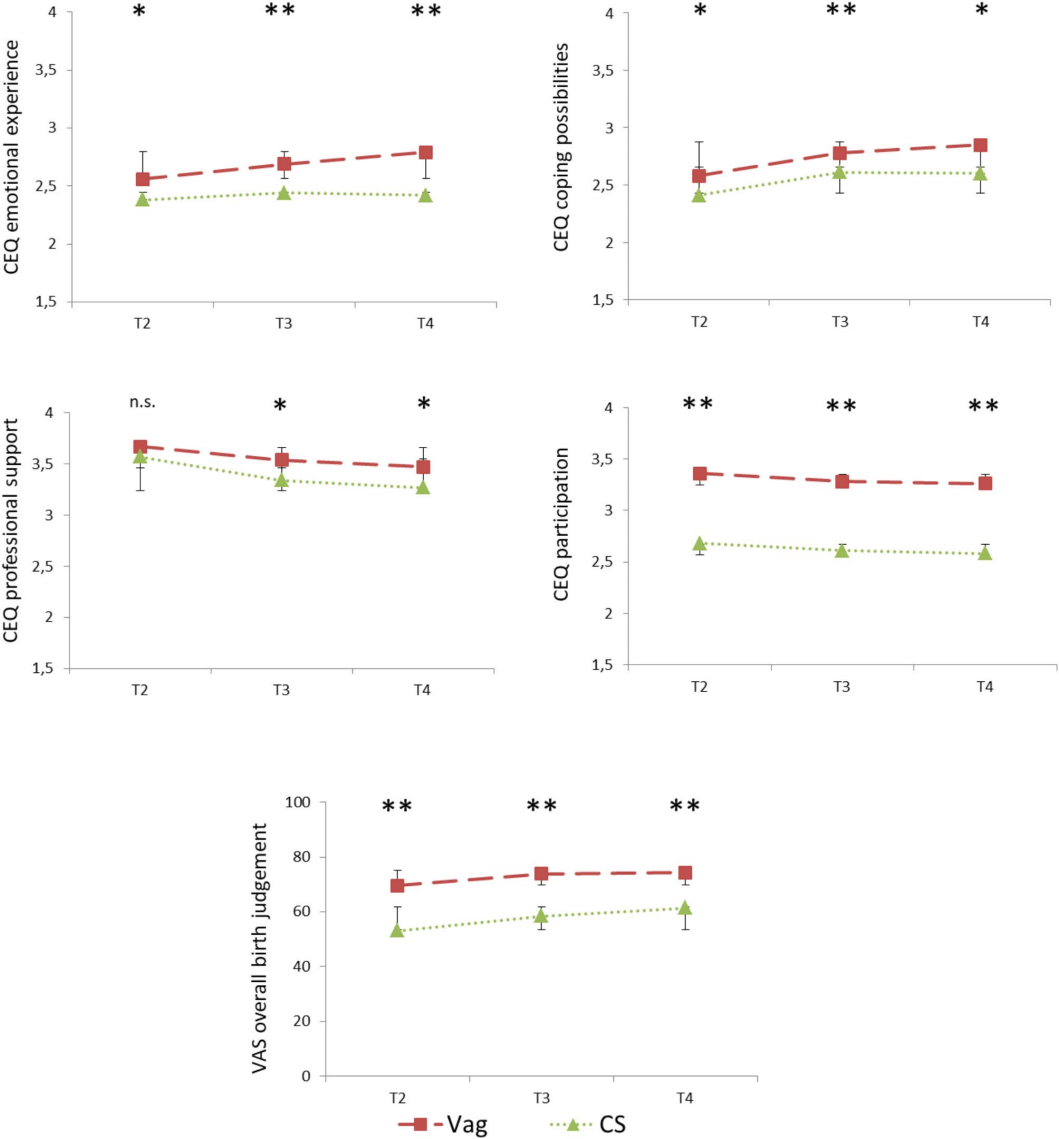
Birthmode and the course of birth experience. Course of the birth assessment (Childbirth Experience Questionnaire and Visual Analogue Scale overall birth judgement) for woman with vaginal birth (Vag) and with caesarean section (CS), with standard deviations. The asterix show the results of the t-tests (two-tailed) for independent samples between both birth modes (*p <.05, ** *p* <.001)

The time courses of the CEQ scales and the VAS *overall birth judgement* depending on the factor FOC (high vs. low) are shown in Fig. 2 and depending on the factor *birth mode* (vaginal birth vs. caesarean section) in Fig. 3. The results of the t-tests for mean differences in the five dependent variables (4 CEQ-scales and VAS overall birth judgement) for every time point can also be found in Figs. 2 and 3.

### Three-way interactions

While no three-way interaction was found for the VAS *overall birth judgement* or for three of the four CEQ subscales (*p* >.310), a three-way interaction was found for the CEQ scale *emotional experience*: *F* (1.75, 320.91) = 4.81, *p* =.012 η_P_^2^ = 0.03. Depending on the FOC and birth mode, women experienced different changes regarding the emotional experience of their birth.

### Two-way interactions

Regarding the twofold interactions, significant interactions were also only found for the CEQ subscale *emotional experience.* There was a significant interaction between *FOC* and *time* (*F* (1.75, 320.91) = 3.88, *p* =.027, η_P_^2^ = 0.02). Women with high FOC showed more constant and lower *emotional experience*-scores (T2: *M* = 2.06 *SD* = 0.69, T3: *M* = 2.00 *SD* = 0.72, T4: *M* = 2.13 *SD* = 0.77) than women with low FOC, whose scores were higher and increased over the survey period (T2: *M* = 2.59 *SD* = 0.73, T3: *M* = 2.62 *SD* = 0.73, T4: *M* = 2.79 *SD* = 0.73).

A significant birth mode*time interaction was also revealed (*F* (1.75, 320.91) = 6.42, *p* =.003, η^2^_P_ = 0.03). While the *emotional experience*-scores for women after a vaginal birth increased in the first six months (T2: *M* = 2.56 *SD* = 0.73, T3: *M* = 2.69 *SD* = 0.75, T4: *M* = 2.79 *SD* = 0.72), the scores for women after a ceasarean were lower overall and stagnate (T2: *M* = 2.38 *SD* = 0.80, T3: *M* = 2.44 *SD* = 0.83, T4: *M* = 2.42, *SD* = 0.84). The interaction between *FOC* and *birth mode* was not significant (*p* =.286). Furthermore there were no significant interaction effects for any of the other CEQ scales or the VAS *overall birth judgement* (*p* >.125).

### Main effects

Different main effects of the 3 examined factors *FOC*, *birth mode* and *time* on the birth experience measured with the CEQ and the VAS *overall birth judgement* were found.

A change over time was found by main effects for the CEQ scales *professional support (F* (1.61, 293.98) = 32.50, *p* <.001, η_P_^2^ = 0.15) and *coping possibilities (F* (1.90, 346.84) = 15.60, *p* <.001, η_P_^2^ = 0.08). The professional support perceived during birth diminishes over time (T2: *M* = 3.65 *SD* = 0.46, T3: *M* = 3.46 *SD* = 0.57, T4: *M* = 3.39 *SD* = 0.62). Post-hoc tests detected significant differences between all three time points (T2 vs. T3: *difference* = 0.17, *p* <.001, T3 vs. T4: *difference* = 0.11 *p* <.001, T2 vs. T4: *difference* = 0.28, *p* <.001). In contrast, the values for perceived coping possibilities increased in the first 6 months: T2: *M* = 2.54 *SD* = 0.70, T3: *M* = 2.74 *SD* = 0.68, T4: *M* = 2.75 *SD* = 0.71. In this case, post-hoc tests could detect differences between T2 and T3 (*difference* = 0.20, *p* <.001) and between T2 and T4 (*difference* = 22, *p* <.001), but not between T3 and T4 (*difference* = 0.03, *p* >.999). The main effect for the CEQ scale *participation* was only marginally significant (*F* (1.73, 317.35) = 2.94, *p* =.062 η^2^_P_ = 0.02) with scores declining over time (T2: *M* = 3.14 *SD* = 0.74, T3: *M* = 3.03 *SD* = 0.80, T4: *M* = 3.00 *SD* = 0.82). However, post-hoc tests showed no evidence for individual differences (*p* >.10). For the last CEQ scale *emotional experience* there was no significant main effect (*p* =.133).

The general assessment on the VAS *overall birth judgement* also revealed a significant main effect of *time*: *F* (1.67, 314.02) = 5.07, *p* =.010, η^2^_P_ = 0.03. The overall assessment of the birth became more positive with increasing time from birth (T2: *M* = 64.51 *SD* = 24.84, T3: *M* = 68.02 *SD* = 26.02, T4: *M* = 70.20 *SD* = 26.10) In post-hoc-tests the measurement times T2 and T3 (*differenc*e = 4.29, *p* =.031) and T2 and T4 (*difference* = 4.97, *p* =.0429) differed from each other in post-high tests; there was no longer any significant change between T3 and T4 (*difference* = 0.69, *p* >.999).

Regarding the main effects of the factor *FOC*, the high-anxiety women differed from the low-FOC-women in all four CEQ scales and the VAS *overall birth judgement*: *emotional experience F* (1, 183) = 20.84, *p* <.001 η^2^_P_ = 0.10, *coping possibilities F* (1, 183) = 30.15, *p* <.001 η_P_^2^ = 0.14, *professional support F* (1, 183) = 10.94, *p* <.001 η_P_^2^ = 0.06 and *participation F* (1, 183) = 4.86, *p* =.029 η_P_^2^ = 0.03. For every CEQ scale the group of women with high FOC (*emotional experience*: *M* = 2.06 *SD* = 0.66, *coping possibilities*: *M* = 2.13 *SD* = 0.51, *professional support: M = 3.22 SD = 0.56*,* participation: M* = 2.82 *SD* = 0.74) showed lower scores than woman with low FOC (*emotional experience*: *M* = 2.70 *SD* = 0.67, *coping possibilities*: *M* = 2.78 *SD* = 0.58, *professional support: M* = 3.56 *SD* = 0.48, *participation: M* = 3.15 *SD* = 0.70). Overall, regardless of time, the VAS *overall birth judgement* (*F* (1, 188) = 16.75, *p* <.001 η_P_^2^ = 0.08) was answered less positively by women with high FOC (*M* = 55.07 *SD* = 22.04) than by women with low FOC (*M* = 70.55 *SD* = 21.51).

Independent-samples t-tests detected significant differences for 14 out of 15 individual comparisons and are shown in Fig. 2. The time courses of the 4 CEQ scales and the VAS *overall birth judgement* as well as the results of the t-test for independent samples (two-tailed) are shown in Fig. 2.

Regarding the main effect *birth mode* the women after a vaginal birth differed from the women after a caesarean section in terms of perceived *participation* measured with the CEQ: *F* (1, 183) = 29.37, *p* <.001, η_P_^2^ = 0.14. Women reported greater participation after a vaginal birth (*M* = 3.30 *SD* = 0.61) than women after a caesarean section (*M* = 2.62 *SD* = 0.71). There was a marginal effect for *professional support*: *F* (1, 183) = 2.80, *p =*.096, η_P_^2^ = 0.02. Women after a vaginal birth tended to report stronger professional support (*M* = 3.56 *SD* = 0.45) than women after a caesarean section (*M* = 3.38 *SD* = 0.61).The other two CEQ scales were not significantly different (*p* >.325). Regarding the VAS *overall birth judgement* a main effect of *birth mode* was also found: *F* (1, 188) = 14.74, *p* <.001, η_P_^2^ = 0.07. Women after vaginal birthgave a more positive overall assessment of the birth (*M* = 72.46 *SD* = 21.17) than women who gave birth by caesarean section (*M* = 58.53 *SD* = 22.18). The time courses of the 4 CEQ scales and the VAS *overall birth judgement* as well as the results of the t-test for independent samples (two-tailed) are shown in Fig. 3.

### Mental health and birth experience

In order to investigate the course of depressive and posttraumatic symptoms of the women over time two one way ANOVAs with the factor *time* (T2, T3 and T4) were calculated with the dependent variables EPDS and IES respectively. For the depressive symptoms, measured with EPDS, a trend was revealed (*F* (1.87, 363.88) = 2.88, *p* =.061, η_P_^2^ = 0.02), while posttraumatic stress symptoms after childbirth, measured with the IES, showed a significant main effect of time: *F* (1.80, 344.34) = 34.92, *p* <.001, η_P_^2^ = 0.16. Post hoc tests detected significant differences between T2 and T3 (*difference* = 3.52, *p* <.001) and T3 and T4 (*difference* = 1.70, *p =*.004) and also the difference between T2 and T4 was significant (*difference* = 5.21, *p* <.001): The degree of posttraumatic symptoms decreased continuously over time between every measurement time point.

Furthermore, pearson correlations calculated between childbirth experience and depressive symptoms (EPDS) as well as posttraumatic symptoms (measured with IES) revealed significant associations at all three postpartum measurement time points. At all time points (T2, T3 and T4), all four CEQ scales and the VAS *overall birth judgement* correlated with posttraumatic symptoms significantly with moderate effects sizes. Weak to moderate correlations were found between CEQ scales and VAS *overall birth judgement* and depressive symptomsat all three postpartum measurement times. Only 3 individual correlations showed non-significant results, see Table 3.

**Table 3:** Correlation between childbirth experience and mental distress

	*N*	CEQ emotional experience	CEQ coping possibilities	CEQ professional support	CEQ participation	VAS birth overall judgement
T2: 2 days p.p.						
EPDS	267	− 0.33**	− 0.34**	− 0.27**	− 0.21**	− 0.35**
IES	265	− 0.41**	− 0.35**	− 0.16*	− 0.17*	− 0.36**
T3: 6 weeks p.p.						
EPDS	248	− 0.17**	− 0.29**	n.s.	− 0.14*	n.s.
IES	247	− 0.38**	− 0.29**	− 0.35**	− 0.31**	− 0.39**
T4: 6 months p.p.						
EPDS	221	− 0.18*	− 0.27**	− 0.19*	n.s.	− 0.20*
IES	219	− 0.51**	− 0.42**	− 0.43**	− 0.38**	− 0.49**

Note: Pearson correlations between childbirth experience and depressive (Edingburgh Postpartum Depression Scale) and traumatic (Impact of Event Scale following childbirth) symptoms, each at the same time point (n.s. not significant, *p <.05, **p <.001)

## Discussion

The aim of the present study was a systematic analysis of different aspects of the subjective birth experience of women aiming to give birth vaginally. The focus was on the change over time, the influence of FOC and birth mode as well as the connection with psychological stress after birth. The birth experience is neither globally nor in its different dimensions a stable experience, but changes in the first 6 months postpartum. FOC was identified as a factor influencing all birth experience dimensions. In contrast, the mode of birth (as a rather objective birth experience) only changed individual aspects of the subjective birth experience. For the *emotional experience* of birth, we determined a complex interaction between *fear of childbirth*, *birth mode* and *time* passed since birth. The significant correlations between the subjective perception of birth and depressive and post-traumatic stress symptoms show the importance of the woman’s perspective on her birth for mental health.

### Influence of time

In terms of the *VAS overall birth judgement*, our data show a small improvement in birth assessment, but with only a small effect size. As Conde and colleagues already pointed out in their results [44], the overall assessment of birth does not change significantly within the first six months. Only with a differentiated look at the various components of the birth experience individual changes can be identified.A significant change in the birth experience is evident in our data for *professional support* and *coping possibilities*, as well as in the global birth assessment. Furthermore the subscale *participation* shows a marginal significant change over time. No significant effect of time could be revealed for emotional experience.

The perceived *professional support* decreases continuously in the first six months after birth. As Turkmen and colleagues already found a reduction for the first three months, the perceived quality of support by midwives and doctors seems to continue to decline over time [45]. Turkmen’s presumed ceiling effect may also have been evident in the present data for T1. The large effect size of our finding also suggests that women evaluate professional support during birth less positively over time.We found a marginal reduction in perceived *participation* during birth. The women in our sample tend to feel less actively involved in the birth process as time passed. Turkmen already found a significant reduction after three months. One reason why this effect became less noticeable in the present study compared to Turkmen could be the significantly higher rate of caesarean deliveries than in Turkmen’s sample, which is discussed in detail under the aspect “influence of birth mode”. Women rated their own coping skills (*coping possibilities)* more positively at 6 weeks than 2 days after birth. However, this increase did not continue - the assessment between 6 weeks and 6 months postpartum no longer differs. The same pattern is found for the general birth assessment *VAS overall birth judgement*: between T2 and T3 the assessment improves, between T3 and T4 there is no significant difference. Both variables seem to already consolidate in the first few weeks after birth.

One possible explanation for the different directions of development in the subscales could be that more internally based birth experiences, such as coping skills and emotional experience, improve over time. In contrast, externally based experiences, such as participation and professional support, deteriorate. This assumption is discussed further below.

Interestingly, the subscale *emotional experience* does not show a significant effect of time. Here the estimations remain stable from 2 days until 6 months after birth. Turkmen used the original evaluation of the CEQ [45]. This has slightly different subscales compared to the German version applied in the surrent study. The subscale *emotional experience* is most comparable to the factor *perceived safety*. In Turkmen’s sample, there is no significant change between one week and 3 months either. As shown in the analysis and discussed in detailed later in the discussion other factors such as birth mode and FOC play a more dominant role on the subscale *emotional experience*.

### Influence of fear of childbirth

The results highlight that the antepartum FOC has a strong influence on the birth experience. Women with strong fear of birth show a more negative assessment on all dimensions of the birth experience and the general birth assessment than women with little or no FOC. All dimensions of the birth experience examined are impaired in women with severe FOC. The significance of FOC for negative birth experiences has already been identified in a meta-analysis based on 18 studies [58]. As the authors emphasize, there are still many unanswered questions as to exactly how risk factors for negative birth experiences work. The present analysis thus adds to the knowledge that FOC affects not only partial aspects, but all dimensions of childbirth To the best of our knowledge this is a new finding, highlighting the strong influence of FOC on the birth experience and the importance for FOC for obstetric care.

With regard to the results on the change of the birth experience over time, the above mentioned explanation that more internally based birth experiences (*coping possibilities* and *emotional experience*) improve over time and in contrast, externally based experiences (*participation* and *professional support*) deteriorate fits well for our data on the time effects as well as for the results of others studies [42, 59]: However, this mechanism does not apply in the same way to women with severe fear of childbirth as the data shows that under the stress of FOC, all aspects of birth appear negatively affected at all time points.

In 2020, Hildingson investigated the effectiveness of an online-based cognitive therapy for women with FOC [60]. The authors show that women with high FOC had a less positive overall assessment of their birth one year postpartum and especially low scores on the subscale of own capacity, i.e. their own coping abilities. A positive effect of cognitive behavioral therapy (vs. midwifery care) which aimed at reducing FOC during pregnancy could not be found. It is possible that the birth experience is crucial in determining how birth anxiety persists after birth. Dencker and colleagues reported in 2018 that the most common cause of FOC in multiparous women is a previous negative birth experience [20]. They emphasize the importance of a negative birth experience for the development of birth anxiety after birth. Because of this result they clearly advocate a distinction between pre-birth and post-birth FOC. It would be useful to examine whether an intervention that focuses on the birth experience instead of or in addition to FOC might lead to different results. It is possible that the role of the subjective birth experience has not been sufficiently considered in the treatment of FOC. In order to prevent a vicious circle from developing in which birth anxious women have negative birth experiences, which in turn lead to further birth anxiety after birth, there is an urgent need to increase attention to women with negative birth experience in clinical practice. To better understand the possible relationships, a similar experimental design could be used to support first-time mothers with FOC with an intervention such as cognitive-behavioural therapy. It is possible that, as with Hildingson, FOC is not reduced. However, it would be interesting to investigate whether the childbearing experience of these women differs from women without therapeutic treatment. In their model of birth satisfaction, Preis and colleagues find that incongruence between the expectation and the actual birth experience decreases birth satisfaction [61]. They therefore clearly recommend that one-to-one conversations and addressing women’s individual needs are necessary to increase satisfaction. Subjective birth expectations and experiences also play a central role here.

Working through the birth experience as one of the potentially anxiety-inducing components could potentially mitigate the long-term effects of a negative birth experience. The women and families affected would benefit considerably from this. In addition, a possible reduction in costs due to desired caesarean sections after stressful birth experiences or the necessary treatment of mental illnesses would relieve the burden on the health system.

### Influence of birth mode

In addition to the influence of FOC, we also analysed the influence of birth mode. The results revealed that the mode of birth changes the birth experience in the subscale of *participation* and the overall assessment VAS *overall birth judgement*.

After a vaginal birth, women report a stronger *participation* during birth and also a more positive overall assessment than women after an unplanned caesarean section. The same pattern emerged for *professional support*, but only as a statistical trend.

As described above, the subjective sense of control may have played a mediating role for the amount of participation. Less active participation during birth could be caused by increased interventions and complications. Similarly, Turkmen and colleagues identified postpartum complications as a predictor for the dimensions *professional support* and *participation*- an increase in complications under or after birth led to a reduction in *participation* and lower reported satisfaction with professional support [45].

Our sample has a caesarean rate of about 30%. In these cases of unplanned caesarean, the women had to terminate the attempt to deliver their baby vaginally for medical reasons, although it was planned otherwise and their aim was to give birth vaginally. The intended expectations of the birth process are therefore not fulfilled. As Lowe pointed out in her analyses 20 years ago [62], self-efficacy in relation for labor plays a significant role. In the case of an aborted attempt for vaginal birth, this often happens on the clear advice of the medical staff. Due to the disparity in experience and knowledge between experts and patients, it is very difficult to perceive this decision as actually having been made jointly. It would be useful to examine more closely how the decision to have a caesarean section was assessed by the women in terms of their participation.

This type of rather unilateral decision-making could therefore lead to a reduction in the women’s sense of participation. This would also explain the less pronounced decline in participation in the first six months in our sample compared to Turkmen’s sample, which only has a caesarean section rate of less than 8%. For example, Hildingson reports that after a spontaneous birth, women rate their own capacity higher than after all other birth modes. Emergency sections in particular have a negative impact on women’s sense of their own competence [60]. In a qualitative survey on decision-making in childbirth from Spain, the authors emphasize the importance of shared decision-making for women’s sense of participation and control [63].

As many other studies have shown, the mode of birth has a considerable influence on the satisfaction with birth experience: The experience of an unplanned caesarean section reduces satisfaction with the birth experience [6, 44, 64–67] and increases the likelihood of a negative birth experience [26]. However, what complements the present analysis, is the particular relevance of subjective expectation (here: the FOC) compared to the objective birth experience (here: birth mode): While the birth mode only changes individual aspects parts of the birth experience with a main effect, FOC influences every single one of the four subscales investigated as well as the global judgement of birth. The finding highlights once more the importance of the woman’s subjective perspective compared to obvious, easily measurable medical parameters. Fenaroli, for example, also highlights the importance of psychological influences on birth satisfaction compared to classical medical parameters [68].

The interactions between FOC, birth mode and timedo not seem to account for any significant variance for the birth assessment, apart from the CEQ subscale *emotional experience*.*Emotional experience* is the only subscale which shows a significant effect of *birthmode* and *FOC*, whereas the main effect time is non-significant. The *emotional experience* differs in that way from the other aspects of the birth experience. The pure temporal course seems to be less important than the interaction between *time* and *birthmode* as well as between *time* and *FOC*. The change in the experience of childbirth thus appears to be influenced primarily by the interaction of the factors of time and birth mode or time and FOC rather than by the time elapsed since birth alone.

Women with low FOC showed higher emotional satisfaction overall, and the score decreased over time. On the other hand, women with high FOC rated the emotional experience lower at all times and the deterioration of the experience was absent.

Women after a vaginal birth showed more positive emotional experiences, which even increase over time. In contrast, women after a caesarean section started with a more negative assessment and remain at that level after 6 months.

This pattern is not the same, but it fits in with the study by Waldenström et al. [69]. They found that negative birth experiences were the cause of deterioration in birth assessment in the first two years postpartum, while women tended to show constant assessment after positive birth experiences within the first two years. The authors suggested that early assessment is colored by the feeling, that birth has finally been mastered, and that negative experiences take longer to be integrated.

Stadlmayr and colleagues were also able to show these negative consequences for women after bad birth experiences in 2007: Many dimensions of birth experiences improve in the first year postpartum. However, women with an overall negative birth experience have a high risk of retaining a negative memory in all seven subscales of the birth experience [42].

The authors reported these results for the general birth assessment and individual subscales. In our study, this result was only available for the subscale *emotional experience*. The *emotional experience* of birth could be a kind of “concentrate” in which the subjective experiences accumulate: External influences are factored out, and the behavioral response is also not taken into account. What remains is a purely emotional response to the stimuli that actually took place.

The present study is not able to answer whether negative birth experiences actually take longer to be processed and integrated. However, our data support the theory that women with positive birth expectations and after a birth mode that goes as planned have a different quality of birth memory than women who had negative expectations and an unplanned birth mode, i.e. after an unplanned caesarean section.

### Birth experience and mental health

Overall, the results discussed so far indicate that the differentiated aspects of the birth experience should also be considered separately. This is also important because the birth experience and the development of psychological symptoms do not appear to be independent. Our data shows that mental health is related to all four scales and the overall rating of the birth experience.Other studies already showed that the prevalence for postnatal depression is not stable in the first year after birth [40], and our data fits well with this pattern. We can also see a relationship between the birth experience and the degree of symptoms for postpartum depression, with a decrease of the strength of association over time.

Our data reveals a relationship between birth experience and depressive symptoms and therefore fits well to already published data. As early as 2001, Saisto and colleagues published a prospective study that highlighted the link between birth satisfaction and postpartum depression [70]. Mohammad and colleagues (2011) used multivariate modelling in a prospective study of 353 women to investigate whether birth experience affects the development of postnatal depression. They were able to show that 9 of the 19 birth experience variables examined accounted for 82% of the variance in postnatal depression [71]. They also showed that the significance of birth experience variables predicting postnatal depression decreased between 6 and 8 weeks and 6 months postpartum.

In a meta-analysis from 2016, Bell and colleagues recommended on the basis of 15 studies that, despite difficult comparability of the results, a negative birth experience could promote postpartum depression [32]. Apart from a few exceptions, the studies on which the review was based on used different measurement instruments for the birth experience. With the CEQ, we used a questionnaire validated in German, which also facilitates comparability with other studies. Furthermore, with the 3 measurement points up to six months postpartum, we represent a larger, very vulnerable period of family formation. Taking our data into account, particular attention should be paid to emotional experience and coping skills, which should be investigated more specifically in future research.

All recorded aspects of the birth experience show a medium negative correlation with the occurrence of traumatic stress symptoms, highlighting that a more negative experience on the subscale are associated with higher levels of traumatic stress symptoms. Garthus-Niegel and colleagues have already been able to show the importance of the subjective birth experience on posttraumatic stress-symptoms [33]. Based on a large cohort study with 1499 women, the subjective birth experience evaluated 8 weeks after childbirth had the strongest association with post-traumatic stress symptoms. Our data suggest that the association between subjective birth experience and posttraumatic stress symptoms persists until 6 months. Maybe the memory becomes more global over time suggesting that individual aspects of the experience can no longer be perceived in such a differentiated way like a few days after birth.

As Carter and colleagues point out in a recent meta-analysis, women are more likely to experience postpartum stress symptoms after an unplanned and especially after an emergency CS [72]. The high number of unplanned CS in our sample may have further strengthened the link between the subjective birth experience and post-traumatic stress symptoms. The present analysis emphasize the statement of previously published studies that women are vulnerable to mental stress symptoms after stressful birth experience.

### Limitations

With amonocentric study at only one clinic, we have a selective choice of study participants who chose to give birth at a university hospital with a nearby paediatric clinic. Women and families with a medium and higher obstetric risk and/or a high need for safety choose to give birth in this centre. Presumably due to the inclusion criterion of the first birth and the university hospital with the highest achievable level of care there is a relatively high rate of caesarean sections. Nevertheless, a quite good generalisability of our results is given by the whole study design such as the quite large sample and the high acceptance among the women who were offered participation. Even if the a priori power calculation has determined the achieved sample size: The factors included and their complex interplay with each other can only be described to a limited extent with the present sample.

For the CEQ in the German validation and factor analysis, factors were not found to be congruent between the German and English versions [46, 73]. Even though the naming and translation suggest a high degree of overlap: The subscale *professional support* has the largest intersection with five overlapping items. This particularity should be taken into account when interpreting the results. At the same time, with the German version of the CEQ, we have used a measurement instrument suitable for the sample, which supports the validity of the results.

Our analysis does not consider the connection between FOC and birth mode as this would go beyond the focus of the present study. Many studies point to a complex interplay of FOC, birth mode and birth-experience [21, 23, 74]. The performed analysis does not take into account for example that the rate of women who are afraid to give birth may have influenced the rate of interventions and thus also caesarean sections. Of the women with FOC more babies (10%) had to be transferred to the paediatric clinic after birth than babies of the women without FOC (3%), which also indicates the connection between FOC and medical complications. However, even with a dependency between both factors, the result remains that FOC is a sensitive and early marker for stress, which also includes (even only possible) medical and physical complications.

Even though some of the variables collected were not normally distributed, a mixed factorial ANOVA was used for the analysis. Previous studies have shown that even massive deviations do not lead to an increase in the alpha error probability with a corresponding sample size [75].

Overall, our analyses were able to show mainly medium to small effect sizes. In contrast, the decline in *professional support* over time, the strong influence of FOC on coping skills and the influence of birth mode on *participation* are characterised by strong effect sizes and should be considered in further studies. The complex interplay between FOC, mental health and coping skills is also not considered in the analysis and should be addressed separately in further studies. In addition to FOC and birth mode, there are other factors that have an influence on the birth experience, such as migration status [76, 77], which could also be taken into account in subsequent studies.

### Implications

As shown, prepartum FOC influences all subscales of the birth experience, in contrast to mode of birth. We thus add another finding to the body of research that highlights that prepartum expectations are a kind of lens through which the actual birth experience is experienced and remembered. With regard to the clinical implications, it would therefore make sense to always supplement the purely somatic perspective with the individual psychological perspective of the woman. It may be very economical to use the woman’s subjective perspective on her own birth as key information for the question of which women may also be at risk of developing psychological distress or attachment disorder in the long term.

We were also able to show, that the timing of the evaluation plays a significant role. As Bell and colleagues published in 2018, birth experience should be surveyed not too early but also not too late, describing a survey after 4 days as very early [27]. Our results support this recommendation because there is much change between day 2 and 6 weeks postpartum. Therefore, when determining a valid subjective birth experience, this time window should ideally not be undercut. It is obvious that there are also more sensible and less optimal time windows for therapeutic interventions to counteract the stresses. More one-to-one conversations are needed and individual needs should be seen to counteract possible consequences of an unprocessed stressful birth experience. Further research would certainly be beneficial in order to gain more understanding of the perceived participation, especially in obstetric decisions during birth.

Future research should also address meaningful therapeutic interventions. It is good to know that the self-assessed coping possibilities with the birth experience are not clearly dependent on the birth mode: women after spontaneous birth or other birth modes assess their coping possibilities similarly. Whether group or individual counselling or other therapeutic tools are appropriate for coping with the birth experience and when to start is also of interest. The potential impact of effective support, especially on the development of a healthy family and as a basis for subsequent pregnancies and births, is of high societal interest.

## Conclusion

Taken together the present study highlights that the subjective birth experience changes over time and that FOC and birth mode influence the subjective birth experience and are two relevant factors for obstetric care. The present analysis adds to the existing knowledge that the individual’s FOC influences all aspects of the later remembered birth experience over at least half a year. In comparison, the objective birth experience such as the mode of birth only partially changes the subjective experience, especially in the more external aspects such as participation and professional support. The final determination of a valid subjective birth experience should favourably not take place too early, and the subjective assessment can change - important also in the case of support - during the first weeks. Furthermore, the study shows that there is an important relationship between subjective birth experience and depressive symptoms, therefore highlighting that the way women experience birth plays a significant role in postnatal mental health. With regard to postpartum depression, more focus should be placed on the emotional experience and coping skills. The long-lasting correlation of subjective birth assessment and traumatic stress symptoms could be an indication of the lasting impact of the birth experience. Further research should investigate whether these correlations are confirmed and persist beyond the first six months. In summary, the subjective perspective should be used more in everyday clinical practice for holistic health care. In terms of prevention, it would make sense not only ethically, but also economically, to record an existing fear of childbirth as well as a stressful birth experience at an early stage and to treat it if possible.

## Electronic supplementary material

Below is the link to the electronic supplementary material.

Supplementary Material 1

## Data Availability

The datasets generated and analysed during the current study are available in the OSF repository: https://osf.io/eytzb/.

## Abbreviations

FOC: Fear of childbirth
CEQ: Childbirth experience questionnaire
CS: Ceasarean section
VAS: Visual analogue scale
FOBS: Fear of birth scale
WDEQ: Wijma delivery expactancy questionnaire
EPDS: Edingburgh postpartum depression scale
IES: Impact of event scale
T1: First measurement time
T2: Second measurement time
T3: Third measurement time
T4: Fourth measurement time
VB: Vaginal birth
p.p.: Postpartum
PTSD: Posttraumatic stress disrorder
