# Supplementary material for: A longitudinal study of the subjective birth experience and the relationship to mental health
Source: BMC Pregnancy Childbirth. 2025 Feb 27;25:216. doi: 10.1186/s12884-025-07348-y (PMC11866607; doi:10.1186/s12884-025-07348-y)
Supplement: Supplementary file 1 — Supplementary Material 1 [file 12884_2025_7348_MOESM1_ESM.pdf]

STROBE Statement—checklist of items that should be included in reports of observational studies

	Item No.	Recommendation	Page/ Line No.	Relevant text from manuscript
**Title and abstract**	1	(*a*) Indicate the study's design with a commonly used term in the title or the abstract	2/ 29	"prospective longitudinal study"
		(*b*) Provide in the abstract an informative and balanced summary of what was done and what was found	2/ 25 - 45	"Methods: In this prospective longitudinal study, 307 first-time mothers, planning to give birth vaginally, were assessed for fear of childbirth at approximately 34 weeks of gestation and for obstetric information. Postpartum birth experience and psychological stress was evaluated at 3 days, 6 weeks and 6 months postpartum using the validated Childbirth Experience Questionnaire which comprises the four dimensions emotional experience, participation, professional support and coping possibilities, and a visual analogue scale for a global birth judgement, supplemented by the Edinburgh postpartum depression scale and the Impact of Event Scale.<br>Results: The individual dimensions of the birth experience changed differently within the first six months. Mixed factorial ANOVAs identified a main effect of fear of childbirth for all four dimensions of the birth experience and the global birth judgment. Mode of birth influenced the dimension participation and the global judgement. For emotional experience, a complex interplay between fear of birth, birth mode and time was revealed. Correlation analyses showed significant associations between the birth experience and, in particular, the psychological distress symptoms resulting from childbirth.<br>Conclusions: Prepartum fear of childbirth affects all dimensions of the subjective birth experience, even after six months. The mode of childbirth, on the other hand, only affects the global birth judgement and participation. The stable correlations between the different dimensions of the birth experience and maternal mental health highlight the importance of the birth experience for clinical practise.."
**Introduction**				
Background/rationale	2	Explain the scientific background and rationale for the investigation being reported	3 and following/ 50 and following	"The experience of birth is of great importance for the health of mother and child. Therefore, the WHO recommendation for "intrapartum care for a positive birth experience" emphasises not only a clinical but also a psychologically safe environment. What constitutes a psychologically safe environment for a birth certainly varies from woman to woman. In order to approach this goal, it makes sense to focus on the subjective birth experience of the woman giving birth. (…)"
Objectives	3	State specific objectives, including any prespecified hypotheses	7/ 152 - 166	"The aim of the present analysis was to systematically examine the different facets of the birth experience in a large sample of women aiming to give birth vaginally and to explore the development of the subjective birth experience over time until 6 months postpartum. Due to the high significance for the birth experience, the influence of FOC was included in the analysis. It is likely that the perceived changes over time in the remembered birth experience are also be influenced by prepartum FOC. In addition, the birth mode was included as a further factor. Because the desire for a sense of control over birth is also significant, the mode of birth may also play a role in the subjective birth experience. In a second step, the connection between the different facets of the birth experience and depressive and traumatic symptoms were examined. Although a link between birth assessment and development of depressive and post-traumatic stress symptoms is suggested by the literature (33), it would be important to understand which aspects of the birth experience are of particular importance in this regard and and how these connections develops over time. The aim is to investigate the change in the birth experience over time and the influence of FOC and birth mode on the birth experience. In addition, the connection between the various birth dimensions and depressive and post-traumatic symptoms will be investigated
**Methods**				

Study design	4	Present key elements of study design early in the paper	8/ 181	"As this is a prospective longitudinal study with four measurement time points."
Setting	5	Describe the setting, locations, and relevant dates, including periods of recruitment, exposure, follow-up, and data collection	10, 11/ 243 - 259	Procedure<br>Women were approached when registering for giving birth at the clinic approximately 6 weeks prior to their expected due date. After the inclusion criteria had been checked and before enrolment, the participants received information about the study and gave their written consent. If the inclusion criteria were not met or at least one exclusion criterion was present, participation was excluded. Afterwards, the women filled out the first questionnaire (T1) in the clinic. About 1-2 days after birth, the participants completed the second questionnaire (T2) on the maternity ward. The maternal and obstetric information was taken from the patient's electronic file shortly after birth. The first two questionnaires were based on paper-pencil. The following measurements were carried out online, 6 weeks (T3) and 6 months (T4) after birth. For the online-survey the online platform SoSciSurvey (56) was used. Participants were contacted via email at the predetermined time points. The email included a personal ID which served as an entry code to the online questionnaire and which enabled us to merge the data of the four time points. They were asked to complete the questionnaire within one week on their technical devices at home. If the questionnaire remained unanswered, the participants were reminded after one week by email and after another week by a text message on their mobile phone. As a thank you for their participation the women received a baby suit.The evaluation of the data and the writing of the manuscript took place at the neighbouring university.
Participants	6	(*a*) *Cohort study*—Give the eligibility criteria, and the sources and methods of selection of participants. Describe methods of follow-up<br>*Case-control study*—Give the eligibility criteria, and the sources and methods of case ascertainment and control selection. Give the rationale for the choice of cases and controls<br>*Cross-sectional study*—Give the eligibility criteria, and the sources and methods of selection of participants	7-8/ 169 - 182	(a) "All women with a minimum age of 18 years, pregnant for the first time without any severe previous illness and who were planning to give birth vaginally were eligible to participate. Furthermore, speaking a sufficient level of the German language was necessary in order to fill out the questionnaire. Participation in the study was offered when registering for birth at the hospital in the last trimester of pregnancy. 398 women, who met the inclusion criteria, were approached at the Clinic for Gynaecology and Obstetrics at the University hospital in Düsseldorf Germany between July 2020 and November 2021. 21 of 398 did not participate because they did not fulfil the inclusion criteria after all (n= 16) or were not interested in participating (n= 5). , 377 participants gave informed written consent prior to participation. Seventy women were excluded during the study progress because they did not fill in the first questionnaire (n= 13), they gave birth at another hospital (n= 35) or they received a planned caesarean section (CS) which was not yet known when women were recruited (n= 22). After excluding these cases the final analysis is based on the sample of 307 data sets. As this is a prospective longitudinal study with four measurement time points, sample sizes vary depending on the time of measurement, as can be seen in Fig. 1"
		(*b*) *Cohort study*—For matched studies, give matching criteria and number of exposed and unexposed<br>*Case-control study*—For matched		n.a.

		studies, give matching criteria and the number of controls per case		
Variables	7	Clearly define all outcomes, exposures, predictors, potential confounders, and effect modifiers. Give diagnostic criteria, if applicable	9, 10/ 195 – 241,	Standardised questionnaires and visual analogue scales (VAS) were used for the study. Prepartum, the participants were given one set of questionnaires (T1: 34th week of pregnancy), postpartum they received the same set of questionnaires at three time points (T2: 2 days, T3: 6 weeks and T4: 6 months after birth).<br>Antenatal measures (T1)<br>In order to receive broad and detailed information on birth anxiety , the Fear of Birth Scale (FOBS) and the Wijma Delivery Expectancy Questionnaire (WDEQ) were used to assess specific pre-birth anxiety. The frequently used WDEQ (48) measures birth expectations with a focus on fears regarding childbirth. It consists of 33 statements about possible sensations or evaluations before and during birth, which can be agreed to on a 6-point-likert scale. The sum score ranges from 0 to 165 points, with higher scores indicating greater fears. The work by Wjima and colleagues suggests three levels: up to 84 points: no significant fear of childbirth, 85 to 99 points: severe childbirth-anxiety, and 100 or more points: phobic childbirth-anxiety (49). The cut-off score of 85 points is highly used in the literature (4), Chronbach's alpha is α=.91. The FOBS includes two VAS (50) with the question "How do you feel about the approaching birth?" and the anchors "calm – worried" and "no fear – strong fear" respectively. Like all VAS they had a line length of 100 mm on which the participant could tick her degree of agreement between the two anchors which were placed on the right and left. A mean score of more than 60 mm was defined as FOC (51) The internal cosistency is α= .91. Demographic data such as maternal age, education level, financial situation and whether the pregnancy resulted after fertility treatment were also collected.<br>Postpartal measures (T2, T3, T4)<br>Birth experience was determined by a one-dimensional global satisfaction using a VAS (VAS overall birth judgement) and by a multidimensional instrument using the Childbirth Experience Questionnaire (CEQ). The German version of the CEQ contains 18 (English original: 22) items with a 4-point-likert-scale (from 1 = totally agree to 4 = totally disagree). The German and the English versions differ in the selection of items used and the subscales determined. For the German version used in this study, subjective birth-experience was measured by calculating the mean scores for the four subscales emotional experience (3 items), coping possibilities (4 items), professional support (8 items) and participation (3 items), whereby higher values stand for a stronger confirmation. Internal consistency (calculated for each timepoint) is good and lies between $.79 \leq \alpha \leq .85$ for emotional experience, $.79 \leq \alpha \leq .85$ for coping possibilities, $.83 \leq \alpha \leq .91$ for professional support and $.64 \leq \alpha \leq .76$ for participation. Additionally, a global assessment of birth (with the anchors negative to positive) was recorded by using a VAS (VAS overall birth judgement).<br>The possible consequences of a stressful birth experience were assessed with the German version of the Edinburgh Postpartum Depression Scale (EPDS) (52, 53) and the Impact of Event Scale (IES) (54, 55). The frequently used EPDS includes 10 items on a 4-point-likert-cale (0-3) such as low mood, feelings of guilt or overwhelm, or thoughts of self-harming behaviour and records a total score between 0-30 points. With 15 items on a 4-point-likert-scale the IES measures whether the birth experience has left post-traumatic stress symptoms, like strong feelings about the memories of the birth, sleep disturbances or avoidance of memory. For both questionnaires, EPDS and IES, higher scores represent more and/or stronger symptoms. The internal consistency (calculated for each timepoint) for the IES is between $.84 \leq \alpha \leq .85$ and for the EPDS α =.83.<br>Obstetric measures<br>Obstetrical information was taken after birth from the medical record. The mode of birth, use of epidural or general anaesthesia, possible induction of labour, amniotomy or micro blood tests sub partu or necessary transfer of the baby to the paediatric clinic were determined.
Data sources/ measure	8*	For each variable of interest, give sources of data and details of methods of assessment	9,10	*See above* (Variables/ 8)

ment		(measurement). Describe comparability of assessment methods if there is more than one group		
Bias	9	Describe any efforts to address potential sources of bias		*n.a.*
Study size	10	Explain how the study size was arrived at	8/ 188 - 193	"To calculate the necessary sample-size, the program G*Power was used (47). An a priori power analysis to calculate the required sample size was based on a mixed factorial ANOVA with an estimated effect size of 0.2, an alpha error of .05, and a power of at least .80, resulting in a required sample size of at least 144 complete participants. The increased final sample size resulted from an originally higher calculation for drop-out due to planned caesarean sections and for drop-out in the later measurement time points.."

Continued on next page

Quantitative variables	11	Explain how quantitative variables were handled in the analyses. If applicable, describe which groupings were chosen and why	11/ 266 – 268, 270 - 272	*See above* (Variables/ 8)<br>"The group determination of women with high and low fear of childbirth was calculated based on the mean of FOBS-scales greater or equal 60mm vs. less 60mm, as Ternström and colleagues have already done.<br>Additionally, in order to investigate the influence of birth mode on birth experience, the two groups vaginal birth (VB: spontaneous parturition and instrumental birth) and unplanned caesarean section (CS) were formed."
Statistical methods	12	(*a*) Describe all statistical methods, including those used to control for confounding<br>(*b*) Describe any methods used to examine subgroups and interactions	11 - 12 / 263 - 290	For the statistical analysis the statistical package IBM SPSS Statistics 27 was used. First, the descriptive statistics of the survey as well as the basic medical data were determined. Chi²-tests were calculated to compare the medical outcome between the women with high and low fear of childbirth. The group determination of women with high and low fear of childbirth was calculated based on the mean of FOBS-scales greater or equal 60mm vs. less 60mm, as Ternström and colleagues have already done (51). There was a strong positive correlation between the two FOC measurement tools evaluated at T1; the WDEQ and the FOBS (Pearson correlation: $r (294) = .62$, $p < .001$), so that in the further analysis FOC was only based on the FOBS (57). Additionally, in order to investigate the influence of birth mode on birth experience, the two groups vaginal birth (VB: spontaneous parturition and instrumental birth) and unplanned caesarean section (CS) were formed.<br>For the main analysis regarding the birth experience, five mixed-factor 2 x 2 x 3 - ANOVAs were calculated with the between-subject factor FOC (high FOC vs. low FOC), the between-subject factor birth mode (VB: vaginal birth vs. CS: caesarean section) and the within-subject factor time (T2: 2 days vs. T3: 6 weeks vs. T4: 6 months postpartum). The four CEQ subscales emotional experience, participation, professional support, coping possibilities and the VAS scale overall birth judgement served as dependent variables respectively for the five ANOVAs. Using t-tests (two-tailed) for independent samples, the groups of women with high vs. low FOC and women after vaginal birth vs. caesarean section are tested for mean differences at the different measurement times. If the sphericity assumption was violated, Greenhouse-Geisser corrected values are reported. Bonferroni-corrected post hoc tests are reported.<br>In the next step, two repeated-measure ANOVAs were calculated to examine the course of depressive symptoms (EPDS) and traumatic symptoms (IES) in order to explore changes over time between T2, T3 and T4. Aditionally, pearson correlations between the subjective birth experience, i.e. the four CEQ scales and the VAS overall birth judgement on the one hand and depressive (EPDS) and posttraumatic symptoms following childbirth (IES) on the other hand were calculated.<br>According to Cohen (57), the limits for the effect size of pearson correlations are .10 (small effect), .30 (medium effect) and .50 (large effect), and the limits for the effect size of ANOVAs were .01 (small effect), .06 (medium effect) and .14 (large effect)."
		(*c*) Explain how missing data were addressed	13/ 294 - 308	"The number of participants varied depending on the time of measurement. 72.3 % (N = 222) of the recruited women completed all time points. T2 questionnaires answered later than 7 days postpartum and T3 and T4 questionnaires answered later than 3 weeks after the first invitation were not included. The absolute number of questionnaires for the four measurement points can be seen in Fig.."
		(*d*) *Cohort study*—If applicable, explain how loss to follow-up was addressed<br>*Case-control study*—If applicable, explain how matching of cases and controls was addressed<br>*Cross-sectional study*—If applicable, describe analytical methods taking account of sampling strategy	12	*See above* (Statistical methods/ 12)
		(*e*) Describe any sensitivity		*n.a.*

		analyses		
**Results**				
Participants	13*	(a) Report numbers of individuals at each stage of study—eg numbers potentially eligible, examined for eligibility, confirmed eligible, included in the study, completing follow-up, and analysed	7, 10, 11/ 169- 172, 243-245, 247- 252	"All women with a minimum age of 18 years, pregnant for the first time without any severe previous illness and who were planning to give birth vaginally in the University hospital in Düsseldorf were eligible to participate. Furthermore, speaking a sufficient level of the German language was necessary in order to fill out the questionnaire."<br>"Women were approached when registering for giving birth at the clinic approximately 6 weeks prior to their expected due date. After the inclusion criteria had been checked and before enrolment, the participants received information about the study and gave their written consent."<br>"Afterwards, the women filled out the first questionnaire (T1) in the clinic. About 1-2 days after birth, the participants completed the second questionnaire (T2) on the maternity ward. The maternal and obstetric information was taken from the patient's electronic file shortly after birth. The first two questionnaires were based on paper-pencil. The following measurements were carried out online, 6 weeks (T3) and 6 months (T4) after birth. For the online-survey the online platform SoSciSurvey (55) was used. Participants were contacted via email at the predetermined time points."
		(b) Give reasons for non-participation at each stage	11/ 245 - 247	"If the inclusion criteria were not met or at least one exclusion criterion was present, participation was excluded."
		(c) Consider use of a flow diagram		*Fig. 1*
Descriptive data	14*	(a) Give characteristics of study participants (eg demographic, clinical, social) and information on exposures and potential confounders	13 / 294 - 302	The number of participants varied depending on the time of measurement. 72.3 % (N = 222) of the recruited women completed all time points. T2 questionnaires answered later than 7 days postpartum and T3 and T4 questionnaires answered later than 3 weeks after the first invitation were not included. The absolute number of questionnaires for the four measurement points can be seen in Fig.1.<br>The final sample consisted of 307 women expecting their first child with a mean age of 32.9 years (SD = 4.4, range: 20 and 49 years). The mean gestational age at T1 was 35 weeks (M = 240.9 days, SD = 14.8 days; range: 28th to 39th week of pregnancy). At birth, the gestational age of the participants was 40 weeks (M = 279.2 days, SD = 10.8 days, range 259 – 295 days)<br>Table 1, Tabel 2
		(b) Indicate number of participants with missing data for each variable of interest		*The underlying sample size in each case is listed in the tables (1, 2 and 3).*
		(c) *Cohort study*—Summarise follow-up time (eg, average and total amount)		*Fig. 1*
Outcome data	15*	*Cohort study*—Report numbers of outcome events or summary measures over time	13, 19	*Table 1 and 3*
		Case-control study—Report numbers in each exposure category, or summary measures of exposure		*n.a.*
		Cross-sectional study—Report		*n.a.*

		numbers of outcome events or summary measures		
Main results	16	(*a*) Give unadjusted estimates and, if applicable, confounder-adjusted estimates and their precision (eg, 95% confidence interval). Make clear which confounders were adjusted for and why they were included		*n.a.*
		(*b*) Report category boundaries when continuous variables were categorized	13/ 303 - 306	19.3 % (N = 58) of the participants showed high FOC with a mean of the FOB scales equal or higher than 60 mm and are assigned to the high FOC-group in the following. The remaining 80.7% (N = 243) women whose scores were below this threshold were assigned to the group with no or low fear of childbirth and were called the low FOC-group in the further course.
		(*c*) If relevant, consider translating estimates of relative risk into absolute risk for a meaningful time period		*n.a.*

Continued on next page

Other analyses	17	Report other analyses done—eg analyses of subgroups and interactions, and sensitivity analyses	19/ 428 - 430	In order to investigate the course of depressive and posttraumatic symptoms of the women over time two one way ANOVAs with the factor time (T2, T3 and T4) were calculated with the dependent variables EPDS and IES respectively.
**Discussion**				
Key results	18	Summarise key results with reference to study objectives	20, 21 / 449 - 459	"The aim of the present study was a systematic analysis of different aspects of the subjective birth experience of women aiming to give birth vaginally. The focus was on the change over time, the influence of FOC and birth mode as well as the connection with psychological stress after birth. The birth experience is neither globally nor in its different dimensions a stable experience, but changes in the first 6 months postpartum. FOC was identified as a factor influencing all birth experience dimensions. In contrast, the mode of birth (as a rather objective birth experience) only changed individual aspects of the subjective birth experience. For the emotional experience of birth, we determined a complex interaction between fear of childbirth, birth mode and time passed since birth. The significant correlations between the subjective perception of birth and depressive and post-traumatic stress symptoms show the importance of the woman's perspective on her birth for mental health."
Limitations	19	Discuss limitations of the study, taking into account sources of potential bias or imprecision. Discuss both direction and magnitude of any potential bias	29, 30/ 663 - 696	"Limitations<br>With amonocentric study at only one clinic, we have a selective choice of study participants who chose to give birth at a university hospital with a nearby paediatric clinic. Women and families with a medium and higher obstetric risk and/or a high need for safety choose to give birth in this centre. Presumably due to the inclusion criterion of the first birth and the university hospital with the highest achievable level of care there is a relatively high rate of caesarean sections. Nevertheless, a quite good generalisability of our results is given by the whole study design such as the quite large sample and the high acceptance among the women who were offered participation. Even if the a priori power calculation has determined the achieved sample size: The factors included and their complex interplay with each other can only be described to a limited extent with the present sample.<br>For the CEQ in the German validation and factor analysis, factors were not found to be congruent between the German and English versions (46, 73). Even though the naming and translation suggest a high degree of overlap: The subscale professional support has the largest intersection with five overlapping items. This particularity should be taken into account when interpreting the results. At the same time, with the German version of the CEQ, we have used a measurement instrument suitable for the sample, which supports the validity of the results.<br>Our analysis does not consider the connection between FOC and birth mode as this would go beyond the focus of the present study. Many studies point to a complex interplay of FOC, birth mode and birth-experience (21, 23, 74). The performed analysis does not take into account for example that the rate of women who are afraid to give birth may have influenced the rate of interventions and thus also caesarean sections. Of the women with FOC more babies (10%) had to be transferred to the paediatric clinic after birth than babies of the women without FOC (3%), which also indicates the connection between FOC and medical complications. However, even with a dependency between both factors, the result remains that FOC is a sensitive and early marker for stress, which also includes (even only possible) medical and physical complications.<br>Even though some of the variables collected were not normally distributed, a mixed factorial ANOVA was used for the analysis. Previous studies have shown that even massive deviations do not lead to an increase in the alpha error probability with a corresponding sample size (75).<br>Overall, our analyses were able to show mainly medium to small effect sizes. In contrast, the decline in professional support over time, the strong influence of FOC on coping skills and the influence of birth mode on participation are characterised by strong effect sizes and should be considered in further studies. The complex interplay between FOC, mental health and coping skills is also not considered in the analysis and should be addressed separately in further studies. In addition to FOC and birth mode, there are other factors that have an influence on the birth experience, such as migration status (76, 77), which could also be taken into account in subsequent studies.
Interpretatio	20	Give a cautious overall	31/ 726 -	"Taken together the present study highlights that the subjective birth experience changes over time and that FOC and birth mode

n		interpretation of results considering objectives, limitations, multiplicity of analyses, results from similar studies, and other relevant evidence	744	influence the subjective birth experience and are two relevant factors for obstetric care. The present analysis adds to the existing knowledge that the individual's FOC influences all aspects of the later remembered birth experience over at least half a year. In comparison, the objective birth experience such as the mode of birth only partially changes the subjective experience, especially in the more external aspects such as participation and professional support. The final determination of a valid subjective birth experience should favourably not take place too early, and the subjective assessment can change - important also in the case of support - during the first weeks. Furthermore, the study shows that there is an important relationship between subjective birth experience and depressive symptoms, therefore highlighting that the way women experience birth plays a significant role in postnatal mental health. With regard to postpartum depression, more focus should be placed on the emotional experience and coping skills. The long-lasting correlation of subjective birth assessment and traumatic stress symptoms could be an indication of the lasting impact of the birth experience. Further research should investigate whether these correlations are confirmed and persist beyond the first six months. In summary, the subjective perspective should be used more in everyday clinical practice for holistic health care. In terms of prevention, it would make sense not only ethically, but also economically, to record an existing fear of childbirth as well as a stressful birth experience at an early stage and to treat it if possible..”
Generalisability	21	Discuss the generalisability (external validity) of the study results	29/ 663 - 669	With amonocentric study at only one clinic, we have a selective choice of study participants who chose to give birth at a university hospital with a nearby paediatric clinic. Women and families with a medium and higher obstetric risk and/or a high need for safety choose to give birth in this centre. Presumably due to the inclusion criterion of the first birth and the university hospital with the highest achievable level of care there is a relatively high rate of caesarean sections. Nevertheless, a quite good generalisability of our results is given by the whole study design such as the quite large sample and the high acceptance among the women who were offered participation.
**Other information**				
Funding	22	Give the source of funding and the role of the funders for the present study and, if applicable, for the original study on which the present article is based		*n/a*

*Give information separately for cases and controls in case-control studies and, if applicable, for exposed and unexposed groups in cohort and cross-sectional studies.

Note: An Explanation and Elaboration article discusses each checklist item and gives methodological background and published examples of transparent reporting. The STROBE checklist is best used in conjunction with this article (freely available on the Web sites of PLoS Medicine at http://www.plosmedicine.org/, Annals of Internal Medicine at http://www.annals.org/, and Epidemiology at http://www.epidem.com/). Information on the STROBE Initiative is available at www.strobe-statement.org.
